# Response criteria in solid tumors (PERCIST/RECIST) and SUV_max_ in early-stage non-small cell lung cancer patients treated with stereotactic body radiotherapy

**DOI:** 10.1186/s13014-018-0980-7

**Published:** 2018-02-27

**Authors:** Cory Pierson, Taras Grinchak, Casey Sokolovic, Brandi Holland, Teresa Parent, Mark Bowling, Hyder Arastu, Paul Walker, Andrew Ju

**Affiliations:** 1 Leo W. Jenkins Cancer Center, 600 Moye Boulevard, Greenville, NC 27834 USA; 2 North Carolina State University, Raleigh, NC 27695 USA; 3 Department of Internal Medicine, 600 Moye Boulevard, Greenville, NC 27834 USA

**Keywords:** NSCLC, Stereotactic body radiotherapy, PERCIST/RECIST, SUV_max_

## Abstract

**Background:**

The purpose of this study was to evaluate the prognostic impact of Positron Emission Tomography Response Criteria in Solid Tumors (PERCIST) and Response Evaluation Criteria in Solid Tumors (RECIST) and of pre- and post-treatment maximum Standard Uptake Value (SUV_max_) in regards to survival and tumor control for patients treated for early-stage non-small cell lung cancer (ES-NSCLC) with stereotactic body radiotherapy (SBRT).

**Methods:**

This is a retrospective review of patients with ES-NSCLC treated at our institution using SBRT. Lobar, locoregional, and distant failures were evaluated based on PERCIST/RECIST and clinical course. Univariate analysis of the Kaplan-Meier curves for overall survival (OS), progression free survival (PFS), lobar control (LC), locoregional control (LRC), and distant control (DC) was conducted using the log-rank test. Pre- and post-treatment SUV_max_ were evaluated using cutoffs of < 5 and ≥ 5, < 4 and ≥ 4, and < 3 and ≥ 3. ∆SUV_max_ was also evaluated at various cutoffs. Cox regression analysis was conducted to evaluate survival outcomes based on age, gender, pre-treatment gross tumor volume (GTV), longest tumor dimension on imaging, and Charlson Comorbidity Index (CCI).

**Results:**

This study included 95 patients (53 female, 42 male), median age 75. Lung SBRT was delivered in 3–5 fractions to a total of 48–60 Gy, with a BED_α/β = 10Gy_ of at least 100 Gy. Median OS and PFS from the end of SBRT was 15.4 and 11.9 months, respectively. On univariate analysis, PERCIST/RECIST response correlated with PFS (*p* = 0.039), LC (*p* = 0.007), and LRC (*p* = 0.015) but not OS (*p* = 0.21) or DC (*p* = 0.94). Pre-treatment SUV_max_ and post-treatment SUV_max_ with cutoff values of < 5 and ≥ 5, < 4 and ≥ 4, and < 3 and ≥ 3 did not predict for OS, PFS, LC, LRC, or DC. ∆SUV_max_ did not predict for OS, PFS, LC, LRC, or DC. On multivariate analysis, pre-treatment GTV ≥ 30 cm^3^ was significantly associated with worse survival outcomes when accounting for other confounding variables.

**Conclusions:**

PERCIST/RECIST response is associated with improved LC and PFS in patients treated for ES-NSCLC with SBRT. In contrast, pre- and post-treatment SUV_max_ is not predictive of disease control or survival.

## Background

Lung cancer is globally the leading cause of death for men and the second leading cause of death for women, with an estimated 1.8 million new cases every year accounting for nearly 13% of all cancer diagnoses [1, 2], with non-small cell lung cancer (NSCLC) accounting for 80–85% of cases. The American Cancer Society estimates that lung cancer in the United States will cause more than 155,000 deaths in 2017 [3]. For patients with early-stage NSCLC (stages IA, IB, IIA), the 5-year survival rate is 49%, 45%, and 30%, respectively [3]. As such, novel diagnostic and interventional approaches have the potential to improve survival rates of patients with NSCLC.

Due to medical comorbidities often related to heavy cigarette use, 25% of early-stage NSCLC (ES-NSCLC) patients are inoperable at presentation [4]. As a result, stereotactic body radiation therapy (SBRT) has emerged as a viable treatment method capable of displaying high local control rates [4]. Overall survival (OS) associated with SBRT has been shown to correlate with the development of distant metastases, emphasizing the need for predictive identification of tumors that demonstrate a potential for both local and distant recurrence [5].

[^18^F]-fluoro-2-deoxy-glucose positron emission tomography with computed tomography (FDG PET/CT) is often used for tumor staging and post-treatment evaluation in early-stage NSCLC. Maximum standardized uptake value (SUV_max_) provides a quantitative approximation of tumor glucose metabolism [5]. Although SUV_max_ has been consistently demonstrated to be predictive of overall survival for surgically treated NSCLC patients [6], existing research is less consistent on the prognostic value of both pre- and post-treatment SUV_max_ with regard to OS for patients receiving SBRT for NSCLC. Several studies have demonstrated an association between pre-treatment SUV_max_ and OS [7–9], while others have not shown a similar correlation [10–12]. Similarly, post-treatment FDG PET/CT is often used to evaluate tumor response, but interpretation of these findings can be difficult due to FDG uptake at the tumor site caused by radiation-induced pneumonitis, inflammation, and fibrosis [13, 14]. In addition, SUV_max_ has been demonstrated to persist [10] or even increase [15] at the conclusion of SBRT, even without evidence of local, regional, or distant failure, possibly due to radiation-induced pneumonitis and fibrosis. As such, the FDG uptake in these situations does not provide clear evidence of metabolic tumor activity. Based on this uncertainty in the literature, the current retrospective study examines the prognostic impact of pre- and post-SBRT SUV_max_, as well as PERCIST/RECIST to assess for potential correlation to clinical disease control.

## Methods

This single institution retrospective review utilized a large cohort of patients receiving relatively consistent FDG PET/CT assessments in conjunction with SBRT for early stage non-small lung cancer (ES-NSCLC). The study population consisted of all patients treated for T1-2aN0M0 NSCLC with SBRT. Tumor stage was determined according to the American Joint Committee on Cancer, 7th edition [16]. The cohort also included patients presenting with pathology suspicious of cancer on biopsy accompanied by clinical history and imaging that was consistent with ES-NSCLC. For all included patients, SBRT was the preferred modality after consensus recommendation provided by a multidisciplinary team of oncologists and cardiothoracic surgeons. Tumor size, tumor histology, smoking status, and smoking pack-years were obtained for each patient. Patients were excluded from the study if they were previously treated with thoracic radiotherapy, presented with simultaneous lung cancers, or had inconclusive, non-suspicious pathology on biopsy. The Institutional Review Board at East Carolina University approved this retrospective review (UMCIRB-15-000410).

SBRT was delivered using the CyberKnife® Robotic Radiosurgery System. Biologically Equivalent Dose (BED) was calculated for each patient assuming an α/β ratio of 10 Gy. All patients were treated in 3–5 fractions to a total of 48–60 Gy with a BED_α/β = 10Gy_ of at least 100 Gy (range 100–151.2). The majority of patients were treated with fiducial tracking with Synchrony® System for tumor motion tracking. Spine tracking was utilized when the tumor was located adjacent to the spine and respiratory motion was deemed negligible. A small number of patients who could not have fiducials placed were treated with Xsight® Lung Tracking System.

The SUV_max_ is a central component of Positron Emission Tomography Response Criteria in Solid Tumors (PERCIST) (17]. PERCIST 1.0 is based on the percentage change seen in SUV markers in pre- and post-treatment PET scans, which yields four classifications of tumor metabolism in response to therapy: complete metabolic response (CMR) – complete resolution of FDG uptake within the measurable target lesion such that it is less than mean liver activity and indistinguishable from surrounding background blood-pool levels with no new FDG-avid lesions; partial metabolic response (PMR) – reduction of a minimum of 30% in target tumor SUV; stable metabolic disease (SMD) – disease other than CMR, PMR, or progressive metabolic disease (PMD); and PMD – 30% increase in FDG SUV or beginning of new FDG-avid lesions typical of cancer [17, 18]. Similarly, Response Evaluation Criteria in Solid Tumors (RECIST) 1.1 compares pre- and post-treatment tumor dimensions to classify tumor foci changes into four categories: complete remission (CR) – absence of tumor foci for at least 4 weeks; partial response (PR) – minimum 30% decline in tumor diameter that lasts a minimum of 4 weeks; stable disease (SD) – tumor response that does not meet PR or progressive disease criteria; and progressive disease (PD) – absolute increase in total tumor diameters of at least 5 mm [17]. In the current study, PERCIST/RECIST values were obtained on subsequent FDG PET/CT scans at follow up appointments. Best-measured radiographic assessment was determined for each patient based on the follow-up FDG PET/CT scan that demonstrated the most robust tumor response to therapy regardless of previous or subsequent scans. PERCIST was used whenever possible, while RECIST was used when PERCIST could not be obtained.

Lobar control (LC), locoregional control (LRC), and distant failures (DF) were evaluated based in part on PERCIST/RECIST and confirmed by clinical or pathologic evidence of progression as the patients were followed in the clinic over time. This study defines LC as the absence of recurrence of tumor within the treated lobe, LRC as the absence of recurrence of tumor within the treated lobe or lymph node basins, and DF as the recurrence of disease outside of the treated lung or in the contralateral lung. LC was repeatedly assessed by subsequent scans in order to assess for true control of disease.

LC, LRC, overall-, progression free-, distant progression free-, and distant metastasis-free survival were estimated by the Kaplan-Meier method. Progression free survival is defined as an absence of clinical evidence of lobar failure, locoregional failure, or death. The log-rank test was used to conduct univariate comparison of survival curves to determine whether SUV_max_ and PERCIST/RECIST criteria influenced outcomes. Pre- and post-treatment SUV_max_ were evaluated using cutoffs of < 5 and ≥ 5, < 4 and ≥ 4, and < 3 and ≥ 3. PFS and OS were calculated from the final SBRT treatment day. ∆SUV_max_, as defined by the change from pre-treatment to post-treatment SUV_max_, was evaluated at various cutoffs. BED was also analyzed to determine whether BED cutoffs of 100 Gy versus > 100 Gy, or of < 110 Gy versus > 110 Gy, were predictive of PERCIST/RECIST criteria.

Univariate logistic regression analyses were conducted to determine whether age, gender, pre-treatment gross tumor volume (GTV), longest tumor dimension on imaging, or Charlson Comorbidity Index (CCI) were predictive of OS, PFS, LC, LRC, or DC. Each factor was assessed at various cutoffs, including the median value of the factor. Cox regression analysis was then performed on each dichotomous variable that demonstrated statistical significance (*p* < 0.05). All statistical calculations were performed using the MedCalc Statistical Software version 15.6.1 [19].

## Results

The current study identified 95 patients with ES-NSCLC who underwent SBRT between April 27, 2009 and April 8, 2015. Patient demographics and tumor characteristics are shown in Table 1. Treatment characteristics are shown in Table 2, while treatment responses are shown in Table 3. Of the total 95 patients, 86 patients had a pre-SBRT PET/CT with a reported pre-treatment SUV_max_. Sixty-one patients had a reported pre-treatment SUV_max_ ≥ 5. Eighty-four patients had post-treatment imaging that allowed for RECIST to be evaluated, while 71 patients had a reported post-treatment FDG PET/CT where SUV and PERCIST could be evaluated.

**Table 1:** Patient & tumor characteristics

Gender	
Female	53 (66%)
Male	42 (44%)
Age at Treatment Outset	
Median (years)	75
Range (years)	51–92
Smoking History	
Former	59 (62%)
Current	29 (31%)
Never	7 (7%)
Pack-Years Smoking	
Median (pack-years)	50
Range (pack-years)	0.75–210
AJCC Stage	
IA	67 (71%)
IB	27 (28%)
IIA	1 (1%)
Gross Tumor Volume	
Median (cm^3^)	9.4
Range (cm^3^)	1.3–193.5
Longest Tumor Dimension	
Median (cm)	2.3
Range (cm)	1.0–5.4
Charlson Comorbidity Index (non-age factored)	
Median	4
Range	2–8
NSCLC Histology	
Squamous	29 (31%)
Adenocarcinoma	35 (37%)
Bronchial Alveolar Cell	2 (2%)
Poorly Differentiated	4 (4%)
Atypical	25 (26%)

**Table 2:** Treatment characteristics

Fraction/Dose	
48 Gy/4f	14 (15%)
50 Gy/4f	5 (5%)
50 Gy/5f	54 (57%)
54 Gy/3f	3 (3%)
60 Gy/5f	19 (20%)
BED-Gy	
Median	100
Range	100–151.2

**Table 3:** Patient responses

Pre-Treatment PET/CT (SUV)	
Patients	86 (91%)
Median SUV	8.4
Range (SUV)	1.5–31.9
Post-Treatment PET/CT (SUV)	
Patients	71 (75%)
Median SUV	3.2
Range (SUV)	1.0–25.5
Time to Post-Treatment PET/CT	
Median (months)	3.1
Range (months)	1.6–26.3
PERCIST/RECIST Response	
Patients	84 (88%)
CMR/CR	69 (82%)
PMR/PR	14 (17%)
SMD/SD	1 (1%)
PMD/PD	N/A
Recurrence	
Patients w/ Recurrence	21 (22%)
Lobar Failure	8 (8%)
Regional Failure	3 (3%)
Locoregional Failure	4 (4%)
Distant Failure	6 (6%)
Overall Survival	
Median (months)	15.3
Range (months)	0.85–70.7

**Fig. 1:**
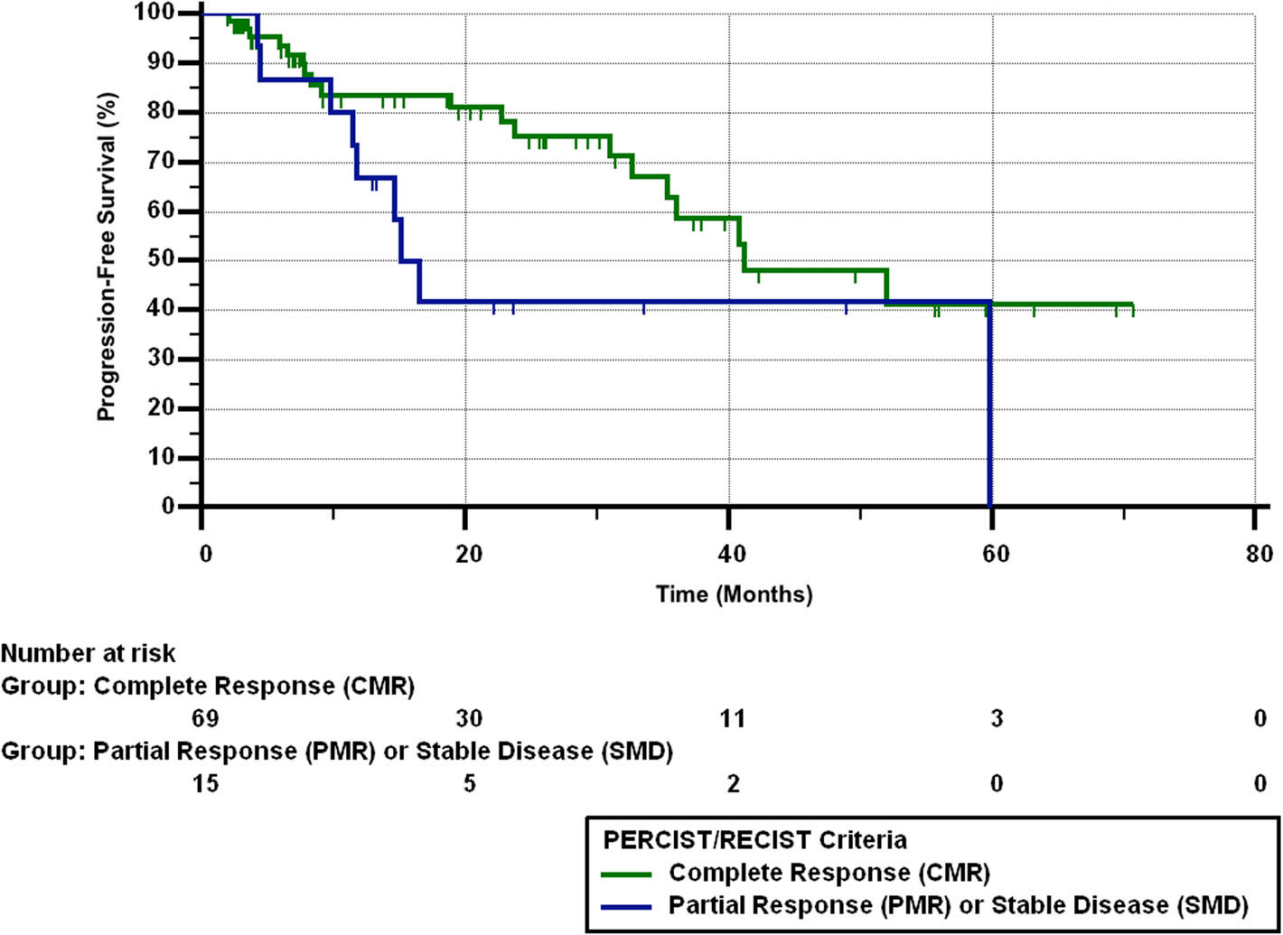
Progression-free survival differentiated by PERCIST/RECIST criteria

**Fig. 2:**
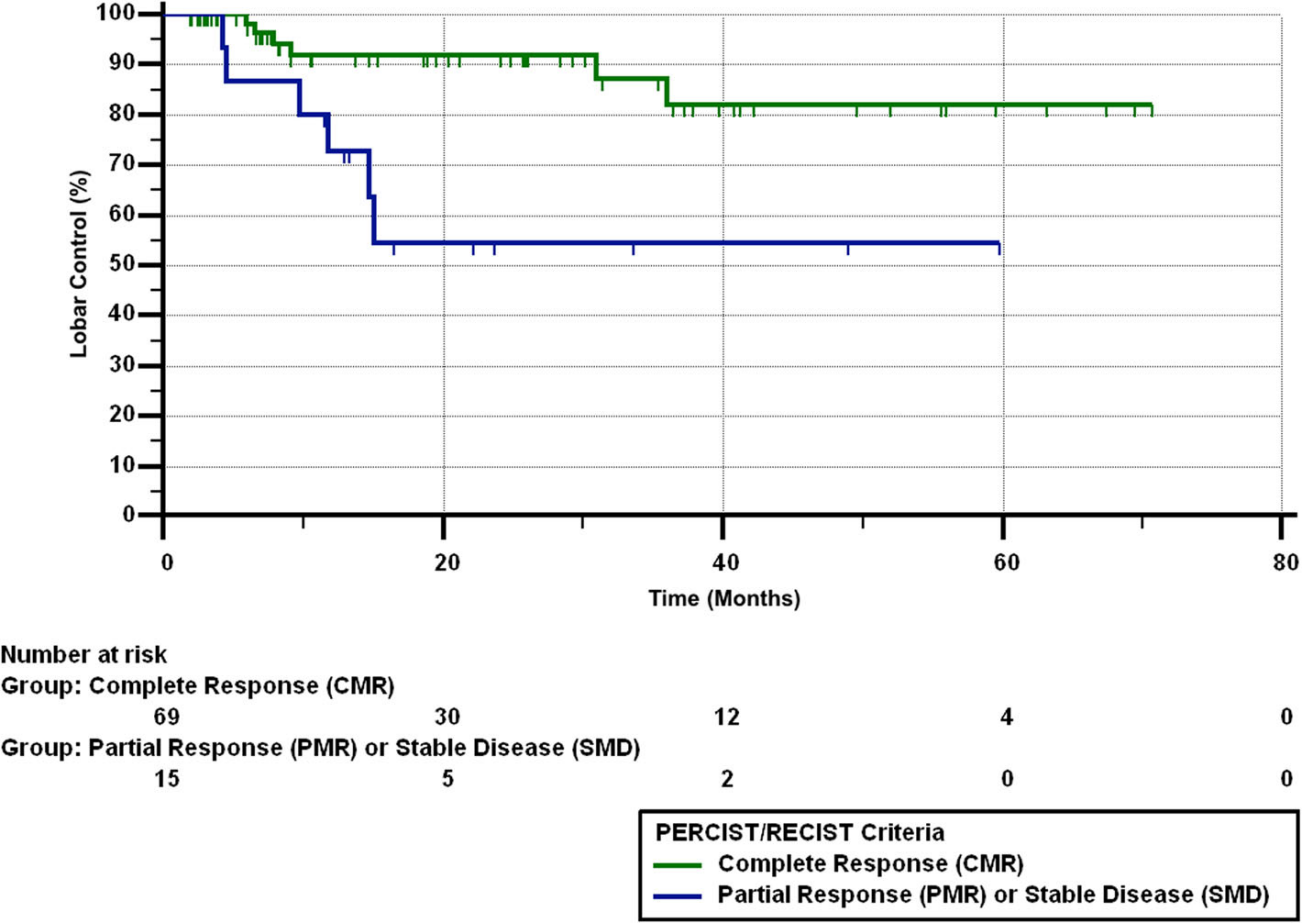
Lobar control rates differentiated by PERCIST/RECIST criteria

**Fig. 3:**
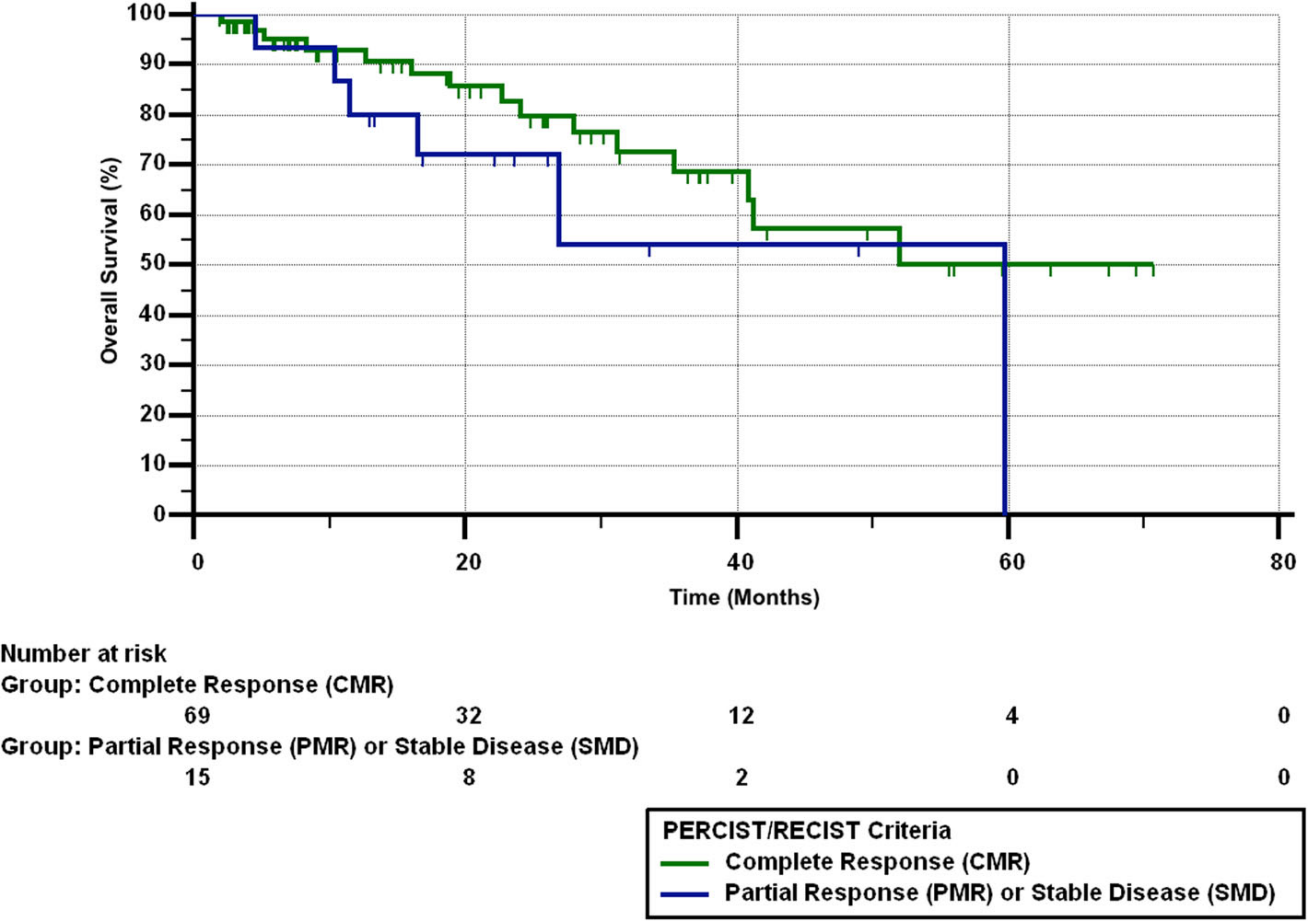
Overall survival differentiated by PERCIST/RECIST criteria

**Fig. 4:**
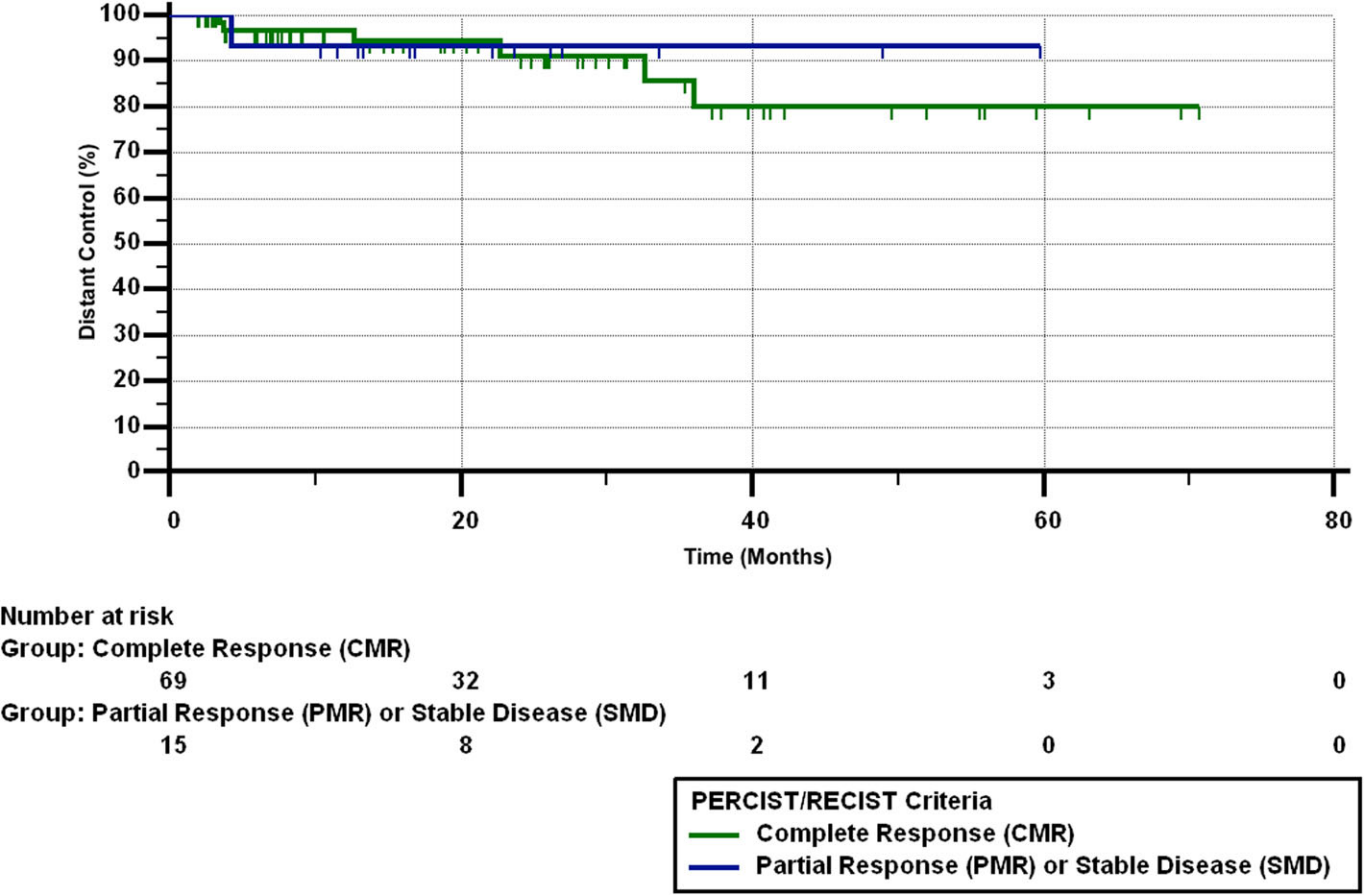
Distant control differentiated by PERCIST/RECIST criteria

Of the 14 patients with a best response of PMR/PR on imaging, 6 had eventual lobar failure as confirmed by clinical course. Of those 6 patients, 1 had locoregional failure, 1 had lobar and distant failure, and 3 died due to lung cancer. Of the remaining 8 patients with PMR/PR, 3 are deceased from other causes and the remaining 5 are alive without disease. Median PFS from the end of SBRT was 11.9 months (range 0.59–70.7 months). Twenty-seven patients (28%) died by the end of the study. Figures 1, 2, 3 and 4 show the progression-free survival, lobar control rates, overall survival, and distant control as differentiated by PERCIST/RECIST criteria.

On univariate analysis, pre-treatment SUV_max_ and post-treatment SUV_max_ with cutoff values of < 5 and ≥ 5, < 4 and ≥ 4, or < 3 and ≥ 3 did not predict for OS, PFS, LC, LRC, or DF. Complete response was predictive of PFS (*p* = 0.039), LC (*p* = 0.007), and LRC (*p* = 0.015), but did not correlate with OS (*p* = 0.21) or DF (*p* = 0.94). BED of 100 Gy versus > 100 Gy, or of < 110 Gy versus > 110 Gy did not predict for PERCIST/RECIST response.

Sixty four patients completed both a pre- and post-treatment SUV_max_, allowing for calculation of ∆ SUV_max_. Median ∆ SUV_max_ was − 5.1 (range = − 26.2 to + 5.9), with a negative value indicating a decrease in SUV_max_ from pre- to post-treatment evaluation. 53 patients (83%) demonstrated a reduction in SUV_max_ after treatment, while 11 patients (17%) demonstrated an increase in SUV_max_. ∆ SUV_max_ was not predictive for OS, PFS, LC, LRC, or DF at any cutoff.

Of the 95 patients in the treatment cohort, 11 patients (11.6%) demonstrated a pre-treatment GTV ≥ 30 cm^3^, which was predictive for OS (*p* < 0.001), PFS (*p* = 0.004), LC (*p* = 0.037), and DF (*p* = 0.015). However, this factor was not predictive for LRC (*p* = 0.29). 5 patients (5.3%) demonstrated a non-age factored Charlson Comorbidity Index (CCI) ≥ 8, which was predictive for PFS (*p* = 0.021) and LC (*p* = 0.003) but not for OS (*p* = 0.11), DF (*p* = 0.63), or LRC (*p* = 0.71). Age, gender, and longest tumor dimension were not predictive for OS, PFS, LC, LRC, or DF at any cutoff.

Multivariate analysis demonstrated that GTV ≥ 30 cm^3^ was still predictive for OS (*p* = < 0.001), PFS (*p* = 0.001), LC (*p* = 0.020), and DF (*p* = 0.006) when accounting for age, gender, and non-age corrected CCI. Similarly, non-age factored CCI was still predictive for LC (*p* = 0.010) when accounting for age, gender, and longest tumor dimension. However, non-age factored CCI was not predictive for PFS (*p* = 0.17) when accounting for age, gender, and longest tumor dimension.

## Discussion

SBRT has emerged as a viable treatment option for patients with medically inoperable NSCLC. Several recent studies have demonstrated clinical outcomes following SBRT as similar to those following lobectomy with systematic lymph node dissection [20, 21]. SBRT has also been associated with local control (LC) rates greater than 90% [22, 23], particularly when delivered with the target planning volume receiving a BED greater than 100 Gy [20]. In a large cohort (*n* = 676), long-term follow-up study of patients treated for ES-NSCLC with SBRT, Senthi et al. found that recurrence was relatively uncommon, with distant failure (DF) being the most frequent and local failure (LF) the least frequent [24]. Data from that study indicated that 12% of patients had DF, 6% of patients had locoregional failure (LRF), and 4% had LF [24]. These findings are consistent with several other patient cohorts in which DF was the most common recurrence pattern. In a patient cohort of 132, Bollineni et al. reported 13% DF and 3.6% LF [14]. In a patient cohort of 95, Horne et al. reported 15.8% DF, 10.5% LRF, and 8.4% LF [25]. In a patient cohort of 72, Burdick et al. reported 26.4% DF and 4.2% LF [5]. In a patient cohort of 57, Satoh et al. reported 30% DF, 21% LRF, and 30% LF [12].

In contrast, LF was the most common recurrence in our cohort with 8% of the patients demonstrating this finding while 6% of our patients showed DF. These disparate findings may be partially explained by our shorter follow-up period (median = 15.3 months) when compared to the follow-up timeframes of the other studies (range = 16–27 months), as there were fewer opportunities for the development or detection of distant metastasis.

Our study defines LF as the recurrence of tumor within the treated lobe and LRF as the recurrence of tumor within the treated lobe or lymph node basins. An informal sampling of similar studies illustrated several different definitions for both local and regional failure. Burdick et al. concluded that a patient had LF when two consecutive CT scans showed increasing lesion size as confirmed by PET imaging with or without positive biopsy for carcinoma [5]. Horne et al. considered a patient to have LF if recurrence was seen within the originally involved lobe or within 2 cm of the initial primary but located outside the originally involved lobe [25]. Hoopes et al. specified regional failure as occurring with lymph nodes > 1.0 cm in the expected anatomic drainage or new PET uptake in a similar location [10]. As such, this variation between studies and institutions when defining local and regional recurrence may serve to complicate any potential comparisons regarding post-treatment tumor progression.

Recent studies regarding the prognostic value of pre- and post-treatment SUV_max_ for patients treated for ES-NSCLC with SBRT have reached varying conclusions. Our analysis indicates that pre- and post-treatment SUV_max_ is not predictive of OS, PFS, LC, LRC, or DF. Hoopes et al. reached a similar conclusion, as their data showed no correlation between pre-treatment SUV_max_ and OS or LC [10]. Other studies have reported that post-treatment SUV_max_ is not predictive for OS [5, 12] or LC [12]. In contrast, several recent studies have shown pre-treatment SUV_max_ to be predictive for overall survival (OS) [25], progression-free survival (PFS) [25, 26], and local control (LC) [27]. Other reports demonstrate that post-treatment SUV_max_ is a reliable predictor for LC [14, 28] and DF [26].

The discrepancy in findings related to SUV_max_ and LC may be partially explained by the presence of radiation-induced pneumonitis. This inflammation and related sequelae seen on imaging may impede adequate assessment of tumor response by clouding the distinctions between residual tumor and necrosis or fibrosis [2, 14]. Therefore, acute radiation pneumonitis may limit the effectiveness of post-treatment FDG-PET/CT by inducing early increases in SUV_max_ and complicating the evaluation of LC following SBRT [13].

The lack of consensus regarding the prognostic value of pre- and post-treatment SUV_max_ may also be influenced by the relative lack of standardization in obtaining an SUV. Marom et al. reports that variation in relative SUV cutoff values, differences in elapsed time between FDG injection and imaging, fasting duration, and blood glucose correction may cause disparity in SUV findings among different institutions [29]. This procedural variation may not allow for direct comparison between studies, as patients with higher SUV_max_ in the current study might have been otherwise categorized with a lower SUV_max_ based on differences in obtaining the pre- and post-treatment SUV.

Although our study failed to demonstrate a correlation between pre- and post-treatment SUV_max_ and treatment outcomes, these findings are noted in the context of a relatively large patient cohort when compared to similar studies. Of the previously cited sources confirming the predictive value of pre- and post-treatment SUV_max_, two studies [27, 28] had smaller patient cohorts (*n* = 85, *n* = 82, respectively), two studies [25, 26] had patient cohorts equal to our study (*n* = 95), and one study [14] had a larger cohort (*n* = 132). By comparison, the three reports [5, 10, 12] that did not find a prognostic component to SUV_max_ had comparatively smaller cohort sizes (*n* = 73, *n* = 58, *n* = 57, respectively). Therefore, we do not believe our negative findings to be a product of inadequate sample size.

The SUV_max_ was evaluated as both a continuous variable and a dichotomous variable using several different cutoff points (e.g. < 3.0 and ≥3.0, < 4.0 and ≥4.0, < 5.0 and ≥5.0) to assess potential correlation with specific treatment outcomes. Satoh et al. also utilized two different cutoff points in their analysis, with one demarcation at < 2.5 and ≥2.5 as well as a separate division at < 5.0 and ≥5.0 [12]. Two studies [14, 25] also utilized a cutoff of < 5.0 and ≥5.0, one study utilized a cutoff of < 4.75 and ≥4.75 [26], and another study used a cutoff of < 6.35 and ≥6.35 [10]. Despite the relative similarity in SUV_max_ cutoffs, the results of these studies still demonstrate varied conclusions regarding the prognostic value of SUV_max_.

Given the relative inconsistency in the literature regarding the predictive value of SUV_max_ using FDG-PET, recent studies have examined the use of [^18^F]-fluorothymidine (FLT) as an additional modality for tracking tumor response and patient outcome [30, 31]. One cohort (*n* = 60) of patients treated for stage I-III NSCLC demonstrated rather unique findings, as superior OS was noted in patients with stable disease on FLT-PET/CT at two-week follow-up while simultaneous FDG-PET/CT was not predictive for OS. As such, the use of FLT-PET/CT might provide a more consistent tool for predicting patient outcome and treatment response when compared to FDG-PET/CT in patients treated for NSCLC.

The retrospective nature of this study presents several inherent limitations, such as the potential for inaccuracies in the medical charts and incomplete or missing information. Several patients in our original cohort were excluded from later analysis because they were lost to follow-up or were unable to obtain approval for post-treatment PET. We recognize that this may have introduced bias. However, we believe our findings to be consistent within the defined subgroups because we were not comparing between patients who did and patients who did not receive post-treatment PET scans. Our study may also have been influenced by non-uniform patient management due to variation in treatment protocols or radiographic interpretation.

## Conclusion

This study demonstrates that while PERCIST and RECIST correlate with PFS, LC, and LRC, pre- and post-treatment SUV_max_, as well as ∆SUV_max_, were not shown to be predictive of OS, PFS, LC, LRC, or DF in patients treated for ES-NSCLC with SBRT. The results regarding pre- and post-treatment SUV_max_ in our study stand in contrast to the results of other recent studies that showed a significant correlation between SUV_max_ and those outcomes. As such, further research regarding the interpretation of pre- and post-treatment SBRT CT/PET scans is needed. Utilizing other SUV_max_ cutoff parameters (e.g. ≥6.0, ≥7.0) would also provide additional data points that might better illustrate the potential relationship between pre- and post-treatment SUV_max_ and specific treatment outcomes.

In addition, the prescribed BED did not correlate with PERCIST/RECIST, indicating the need for further research regarding whether underdosing of the tumor leads to partial response instead of complete response. Since only 1 of the 14 patients with partial response had distant failure, providing chemotherapy post-SBRT may not be indicated for these patients. However, because 6 of the 14 patients with partial metabolic response had local failure, additional ablative techniques, such as a wedge resection, may be beneficial for patients with PERCIST/RECIST partial response.

## Abbreviations

BED: Biological effective dose
CMR/CR: Complete metabolic response
DF: Distant failure
FDG PET/CT: [^18^F]-fluoro-2-deoxy-glucose positron emission tomography/computed tomography
FLT PET/CT: [^18^F]-fluorothymidine positron emission tomography/computed tomography
LC: Lobar control
LRC: Locoregional control
NSCLC: Non small cell lung cancer
OS: Overall survival
PERCIST: Positron emission tomography response criteria in solid tumors
PFS: Progression-free survival
PMD/SD: Progressive metabolic disease
PMR/PR: Partial metabolic response
RECIST: Response evaluation criteria in solid tumors
SBRT: Stereotactic body radiotherapy
SMD/SD: Stable metabolic disease
SUV_max_: Standardized uptake value
